# Psychometric properties of the 52-, 25-, and 10-item English and Spanish versions of the Social Problem-Solving Inventory-Revised

**DOI:** 10.3389/fpsyg.2023.1213784

**Published:** 2023-09-21

**Authors:** Sasja A. Schepers, Sean Phipps, Katie A. Devine, Robert B. Noll, Diane L. Fairclough, Michael J. Dolgin, Kathleen A. Ingman, Nicole M. Schneider, Megan E. Voll, Martha A. Askins, Olle Jane Sahler

**Affiliations:** ^1^Princess Máxima Center for Pediatric Oncology, Utrecht, Netherlands; ^2^St. Jude Children’s Research Hospital, Memphis, TN, United States; ^3^Rutgers Cancer Institute of New Jersey, New Brunswick, NJ, United States; ^4^University of Pittsburgh School of Medicine, Pittsburgh, PA, United States; ^5^Colorado School of Public Health, Aurora, CO, United States; ^6^Department of Behavioral Sciences, Ariel University, Ariel, Israel; ^7^Children’s Hospital Los Angeles, Los Angeles, CA, United States; ^8^Texas Children’s Hospital, Houston, TX, United States; ^9^The University of Texas MD Anderson Cancer Center, Houston, TX, United States; ^10^University of Rochester Medical Center, Rochester, NY, United States

**Keywords:** assessment, caregivers, pediatric oncology, problem-solving, psychometrics

## Abstract

**Objective:**

The Social Problem-Solving Inventory-Revised (SPSI-R) is a widely used instrument to assess problem-solving ability. This study examined the factor structure of the 52-, 25-, and 10-item versions of the SPSI-R and assessed factorial invariance across English- and Spanish-speaking participants. In addition, the internal consistency, test-retest reliability and sensitivity to detect change in problem-solving skills over time were assessed across the three different versions of the SPSI-R.

**Methods:**

Data from three randomized controlled trials, in which caregivers of children with cancer (*N* = 1,069) were assigned to either a problem-solving skills intervention (*N* = 728) or a control condition (*N* = 341), were combined. The SPSI-R was administered at baseline (T1) and immediately post intervention (T2). Reliability and multigroup analyses were performed with confirmatory factor analysis (CFA). Sensitivity to change analyses were performed using repeated measures ANOVA.

**Results:**

Confirmatory factor analysis at T1 showed good fit statistics and internal consistency for the 52- and the 25-item versions, but not for the 10-item version. Factorial invariance was demonstrated across time (T1-T2) and language (Spanish-English) for both the 52- and 25-item versions. Adequate sensitivity to change over time was shown.

**Conclusion:**

The 52- and 25-item versions of the SPSI-R appear reliable and valid for assessment of problem-solving skills in English- and Spanish-speaking caregivers of children with newly diagnosed cancer. The 25-item SPSI-R can be used as a short version measuring problem-solving ability; the 10-item version cannot be considered a reliable measure for this population.

## Psychometric properties of the 52-, 25-, and 10-item versions of the Social Problem-Solving Inventory-Revised

Problem solving is described as “the self-directed cognitive-behavioral process by which a person attempts to identify or discover effective or adaptive solutions to problems encountered in everyday living” (D’ Zurilla and Nezu, 1999). Numerous studies have shown that problem-solving ability is associated with adjustment outcomes (D’ Zurilla et al., 1998; Elliott, 1999; Dreer et al., 2005a,b; Jaffee and D’ Zurilla, 2009). For example, good problem-solving skills (e.g., rational problem solving and positive problem orientation) are associated with fewer physical symptoms (Elliott and Marmarosh, 1994), higher life satisfaction (Dreer et al., 2005a), and better adjustment and social competence (Heppner and Anderson, 1985; Nezu, 1985; Cheng, 2001). Less constructive problem solving is associated with depression, anxiety, and emotional distress (Miner and Dowd, 1996; Cheng, 2001; Kurylo et al., 2004; Dreer et al., 2005a). Similarly, interventions that successfully improve problem-solving skills have been shown to reduce symptoms of anxiety, depression, and posttraumatic stress in people with chronic mental or medical health conditions; (Nezu et al., 1998, 2003; Perri et al., 2001; Ciechanowski et al., 2004; Klein et al., 2011; Ghahramanlou-Holloway et al., 2012) and in their caregivers (Bucher et al., 1999; Sahler et al., 2002, 2005, 2013; Cameron et al., 2004).

The comprehensive 52-item Social Problem-Solving Inventory-Revised (SPSI-R) (D’ Zurilla et al., 2002) is a widely used measure for interventions that target problem-solving ability (Varni et al., 1999; Cameron et al., 2004; Ciechanowski et al., 2004; Askins et al., 2009; Iobst et al., 2009; Klein et al., 2011; Sahler et al., 2013). The SPSI-R consists of five scales: Positive Problem Orientation (PPO); Negative Problem Orientation (NPO); Rational Problem Solving (RPS); Impulsive/Carelessness Style (ICS); and Avoidance Style (AS). The RPS scale can be divided further into four subscales: Problem Definition and Formulation; Generation of Alternative Solutions; Decision Making; and Solution Implementation and Verification. A shorter 25-item version of the SPSI-R measures the same five problem-solving scales but does not divide the Rational Problem-Solving Scale into subscale scores (D’ Zurilla et al., 2002). Finally, a 10-item version of the SPSI-R, consisting of one total problem-solving scale score, was developed by Dreer et al. (2009).

The 52-item SPSI-R domains exhibit adequate to excellent internal consistency for numerous populations and settings (D’ Zurilla et al., 2002; Askins et al., 2009; Iobst et al., 2009; Jaffee and D’ Zurilla, 2009; Klein et al., 2011; Wang et al., 2013). The 52-item version has been translated and validated for the Spanish-speaking population (Maydeu-Olivares et al., 2000) and has been culturally validated for the Hispanic population in the United States (De La Torre et al., 2010). Although it has been used to a lesser extent, the 25-item SPSI-R maintains satisfactory internal consistency in several populations (D’ Zurilla et al., 2002; Cameron et al., 2004; Hawkins et al., 2009; Li et al., 2016). The factor structure has been studied for both the 52-item (Maydeu-Olivares and D’ Zurilla, 1996; D’ Zurilla et al., 2002; Wang et al., 2013) and the 25-item (D’ Zurilla et al., 2002; Hawkins et al., 2009; Li et al., 2016) versions. Most studies confirmed the five-factor structure (Maydeu-Olivares and D’ Zurilla, 1996; D’ Zurilla et al., 2002; Hawkins et al., 2009), with only a few exceptions (Wang et al., 2013; Li et al., 2016). Maydeu-Olivares et al. (2000) replicated the 5-factor structure for the Spanish 52-item SPSI-R and showed language factorial invariance (i.e., equal factor loadings between the two language groups) across all domains, except for the impulsivity/carelessness scale. The 10-item version of the SPSI-R was developed in a single study of three clinical samples of adults (Dreer et al., 2009). Dreer et al. (2009) reported an acceptable person separation reliability [i.e., a measure like Cronbach’s alpha (α)] of 0.72 and demonstrated equivalency between the 10-item and 25-item SPSI-R versions. However, no studies have simultaneously assessed the psychometric properties of the 52-, 25-, and 10-item SPSI-R. In addition, research on the sensitivity to detect change is limited. Sensitivity to change is a critical property to evaluate because the SPSI-R is often used in intervention research. A widely used method to assess a measure’s ability to detect change involves randomized trials in which interventions of known effectiveness are compared with placebo or alternative approaches (Stratford and Riddle, 2005).

As far as we know, no other studies have looked at the psychometric properties of the 25- or 10-item SPSI-R version across different languages (i.e., Spanish and English). The goal of the current study was to examine the factor structure and factorial invariance of the 52-, 25-, and 10-item versions of the SPSI-R across English- and Spanish-speaking caregivers of children with cancer. Next, we tested (a) internal consistency, (b) test-retest reliability, and (c) sensitivity to detect changes in problem-solving skills over time for the 52-, 25-, and 10-item versions of the SPSI-R using real-world data from three randomized clinical trials of a problem-solving skills training intervention (in these specific cases, Bright IDEAS; Sahler et al., 2005, 2013; Phipps et al., 2020). These data inform whether study participant burden could be decreased by using shorter versions of the SPSI-R in English- and Spanish-speaking caregivers of children with cancer without losing critical effect data.

## Methods

### Participants

We analyzed data from three consecutive randomized controlled multicenter trials of Problem-Solving Skills Training (PSST) in caregivers of children with newly diagnosed cancer of any form. The first trial (response rate 75%) evaluated the efficacy of face-to-face PSST (*n* = 186) compared with the efficacy of usual psychosocial care (UPC, *n* = 183) in a sample of 369 caregivers (Sahler et al., 2005). The second trial (response rate 54%) evaluated the specificity of the PSST intervention (*n* = 246) compared with that of non-directive support (NDS, *n* = 158) in a sample of 404 caregivers (Sahler et al., 2013). The third study was a non-inferiority trial (response rate 66%) comparing an online version of PSST (*n* = 324) with the face-to-face intervention (*n* = 296) in a sample of 620 caregivers (Phipps et al., 2020). The most frequent reasons for refusal across the trials were: lack of time/scheduling problems, feeling overwhelmed, and not interested. Participants and non-participants did not differ in language, age, gender, or child’s diagnosis. There was a difference for time since diagnosis within the most recent trial, with participants recruited earlier than decliners (Phipps et al., 2020). Caregivers in all three trials completed the 52-item SPSI-R within 4 to 16 weeks after cancer diagnosis and before randomization (T1) and at 8 to 12 weeks later, at the end of the PSST intervention (T2).

The SPSI-R was completed by 1,069 participants at T1, and complete T1-T2 data were available for 822 caregivers (76.9%; Table 1). We did not observe any between-group differences among any of the background characteristics, except for caregiver sex (*p* < 0.001). Fathers did not participate in the comparison group because the comparison group was derived from trial 1 (UPC) and trial 2 (NDS), which included only mothers. In the third trial, face-to-face PSST was compared with an online version of the intervention to test the hypothesis that the online version would be non-inferior to the face-to-face version. Given the unclear anticipated effect of the online version, we excluded the online intervention group from the present analyses.

**TABLE 1:** Background characteristics of caregivers of children with cancer.

Variable	Full sample (*N* = 1,069)		Intervention (PSST, *N* = 728)		Control (UPC/NDS, *N* = 341)	
	***N***	***%***	***N***	***%***	***N***	***%***
Sex						
Male	41	3.8	41	5.6	0	0.0
Female	1,028	96.2	687	94.4	341	100.0
Age, years (mean ± SD)	36.42	8.31	36.17	8.52	36.95	7.84
Language						
English	826	77.3	576	76.1	250	67.9
Spanish	243	22.7	152	20.1	91	24.7
Highest grade in school (mean ± SD)	13.03	3.67	13.27	3.67	12.52	3.61

NDS, non-directive support; PSST, problem-solving skills training; SD, standard deviation; UPC, usual psychosocial care.

### Procedures

The institutional review board at each of the participating centers approved each of these studies. After providing written informed consent, participants completed the T1 measures and were randomly assigned to face-to-face PSST, UPC, or NDS—depending on the trial (Sahler et al., 2005, 2013). The PSST intervention, Bright IDEAS, which has been described in detail in earlier publications (Sahler et al., 2005; Sahler et al., 2013), consists of six to eight 1-h face-to-face sessions delivered by trained research assistants who had graduate education in psychology or training in behavioral health interventions. Spanish-speaking caregivers received interventions from bi-lingual Spanish-speaking research assistants. A $25 gift card was provided after questionnaire completion at T1 and again at T2.

### Measures

Sociodemographic questionnaire. A sociodemographic questionnaire was used to collect information on patient diagnoses (e.g., type and weeks since diagnosis) and caregiver sex, age, language, and highest completed grade.

Social Problem-Solving Inventory-Revised. The SPSI-R (D’ Zurilla et al., 2002) is a 52-item measure of problem-solving abilities. The 25- and 10-item versions consist of a subset of items from the 52-item version. The SPSI-R 52- and 25-item versions both measure five dimensions of problem-solving: PPO, NPO, RPS, ICS, and AS. Items are rated on a 5-point Likert-type scale ranging from 0 (i.e., “not at all true of me”) to 4 (i.e., “extremely true of me”). Higher scores on each dimension imply a greater intensity of that dimension. The SPSI-R 52- and 25-item versions also yield an overall problem-solving ability score. The 10-item version (Dreer et al., 2009) includes two items from each of the five domains and yields an overall summary problem-solving ability score, ranging from 0 to 40. Excellent internal consistency has been reported for both the 52- and 25-item versions of the total SPSI-R scale in both young adults (i.e., ages 17–39 years; 52-item α = 0.95, 25-item α = 0.89) and middle-aged adults (i.e., ages 40–55 years; 52-item α = 0.96, 25-item α = 0.93). The Cronbach’s α for the five domains of problem solving ranged from α = 0.76–0.95 for the 52-item SPSI-R and α = 0.76–0.89 for the 25-item SPSI-R (D’ Zurilla et al., 2002). In terms of construct validity, a five-factor structure of the SPSI-R was revealed, and factor loadings support this structure (D’ Zurilla et al., 2002). Studies performed in different subcultures have yielded similar results for the construct validity of the SPSI-R. Finally, good concurrent validity was found (D’ Zurilla et al., 2002) between the SPSI-R and the Problem-Solving Inventory (PSI; Heppner and Peterson, 1982).

### Statistical analyses

All participants completed the 52-item version of the SPSI-R. This version includes all items in the shorter 25- and 10-item versions. Mplus version 7.4 was used to perform multigroup confirmatory factor analysis. SPSS version 22 was used to perform sensitivity to change analyses.

### Internal consistency, test-retest reliability, and language invariance

Our analyses focused on reproducing the established 5 factors for the 52- and 25-item versions and one total factor for the 10-item version at T1 for English- and Spanish-speaking caregivers. The fit statistics used to evaluate model fit (Hu and Bentler, 1999; Kline, 2005) were root mean square error of approximation (RMSEA; <0.05 excellent fit, <0.08 adequate fit), comparative fit index (CFI; ≥0.95 excellent fit, ≥0.90 adequate fit) and the weighted root mean square residual (WRMSR; <0.1 excellent fit). Multigroup CFAs were conducted to assess configural (pattern of free and fixed parameters is the same), metric (relative factor loadings are proportionally equal across groups), and scalar (relative indicator means are proportionally equal across groups) invariance across time (T1 and T2) and language (English and Spanish). Usually, a CFI-difference of < 0.01 is considered acceptable in demonstrating measurement invariance (Chen, 2007).

To assess the internal consistency of the different versions of the SPSI-R, we performed analyses for all participants at T1 (*N* = 1,069). Cronbach’s α were calculated according to the average inter-item correlation (Cronbach, 1951). Cronbach’s α values ≥ 0.70 were regarded as satisfactory and ≥ 0.80 as good (Nunnally and Bernstein, 1994). We assessed test-retest reliability across T1-T2 data for the different versions of the SPSI-R with Pearson correlation coefficients (*r*) for all participants assigned to comparison groups (i.e., those not receiving a problem-solving intervention) who had complete data at T1 and T2 (*N* = 279), as well as separately for English (*N* = 204) and Spanish-speaking (*N* = 75) comparisons. Pearson’s *r* values of 0.10 were considered small 0.30 moderate, and 0.50 high (Cohen, 1988). Fisher r-to-z transformation was used to assess the significance of the difference between the correlations of the different versions of the SPSI-R and between the correlations for English- and Spanish-speaking caregivers.

To assess sensitivity to change over time, a repeated measure analysis of variance (ANOVA) was performed for each SPSI-R domain for participants with complete data at T1 and T2 assigned to face-to-face PSST or the comparison group (*N* = 822). Note that we included only the 296 caregivers who were assigned to the face-to-face intervention in the sensitivity to change analyses because the trial of solely online PSST was ongoing at the time of the current study and the degree of efficacy of the online intervention had not been established. As PSST was shown to be an effective intervention in our studies (Sahler et al., 2005, 2013; Askins et al., 2009), we hypothesized that the PSST group, on average, would exhibit better problem-solving skills (i.e., increase more in SPSI-R total, PPO and RPS scores and decrease more in NPO, ICS, and AS scores) than would the UPC/NDS comparison group. Partial eta squared (η^2^) was used as a measure of effect to determine the magnitude of the difference in change over time between the PSST and comparison group. Effect sizes of 0.02 were considered small 0.13 medium, and 0.26 large (Cohen, 1988).

## Results

Two separate CFA’s (one for the 52-item version and one for the 25-item version) were conducted in Mplus version 7.4 on the five *a priori* factors of problem-solving. Another CFA was conducted on the 10-item version, with one *a priori* defined total factor of problem-solving skills. The CFA’s for the 52- and 25-item versions showed adequate model fit, whereas the CFA for the 10-item version showed insufficient model fit (Table 2). Therefore, subsequent analyses were only performed with the 52- and 25-item version. Multigroup CFA’s revealed metric and scalar invariance (i.e., ΔCFI < 0.01) across time (T1 versus T2) and language (English versus Spanish) for the 52- and 25-item versions of the SPSI-R (Table 2). The 52- and 25-item versions of the SPSI-R showed sufficient to excellent internal consistency (α = 0.71–0.94; Table 3) for the total scale and all its subscales. The test-retest reliability of the 52- and 25-item SPSI-R versions was stable over time (*r* = 0.52–0.77; Table 4). Both versions of the SPSI-R detected significant changes in problem-solving skills over time between participants assigned to the problem-solving intervention (PSST) and control (UPC/NDS) groups (Table 4). Participants in the intervention group demonstrated significantly higher changes in their problem-solving skills from T1-T2 on all SPSI-R domains than did participants in the control group (*p* < 0.00–*p* < 0.05), except for the impulsivity/carelessness subscale (*p* = 0.153–0.132).

**TABLE 2:** Confirmatory factor multigroup analysis for the 52-, 25-, and 10-item version of the SPSI-R.

	Items	Factors	χ ^2^	df	*p*	RMSEA	WRMR	CFI	ΔCFI
CFA Model Fit Across Measure Versions	52	5	7655.75	1.264	0.00	0.06	2.55	0.91	
	25	5	2162.72	265	0.00	0.07	2.18	0.93	
	10	1	1542.66	35	0.00	0.18	3.70	0.73	
**Measurement Invariance**									
*SPSI-R 52 items*									
**Time point (T1 vs. T2)**									
Configural Invariance*			11506.59	2.528	<0.001	0.06	3.14	0.92	*na*
Metric Invariance**			11342.52	2.575	<0.001	0.06	3.14	0.92	0.001
Scalar Invariance***			11413.85	2.726	<0.001	0.06	3.18	0.93	0.001
**Language (English vs. Spanish)**									
Configural Invariance			7617.91	2.528	<0.001	0.05	2.73	0.93	*na*
Metric Invariance			7555.84	2.575	<0.001	0.05	2.74	0.93	0.002
Scalar Invariance			7757.81	2.726	<0.001	0.05	2.81	0.93	0.001
*SPSI-R 25 items*									
**Time point (T1 vs. T2)**									
Configural Invariance			3064.46	530	<0.001	0.07	2.59	0.94	*na*
Metric Invariance			2971.15	550	<0.001	0.07	2.61	0.94	0.003
Scalar Invariance			2992.78	620	<0.001	0.06	2.66	0.94	0.001
**Language (English vs. Spanish)**									
Configural Invariance			2240.34	530	<0.001	0.07	2.37	0.94	*na*
Metric Invariance			2230.13	550	<0.001	0.07	2.41	0.94	0.001
Scalar Invariance			2398.25	620	<0.001	0.06	2.54	0.93	−0.004

CFA, confirmatory factor analysis; CFI, comparative fit index; df, degrees of freedom; CFI ^Δ^, difference in CFI; *p*, *χ*^2^ *p*-value, RMSEA, root mean square error of approximation; WRMR, weighted root mean square residual; *χ*^2^, model chi square; *the pattern of fixed and free parameters is the same; **the relative factor loadings are proportionally equal across groups; ***the relative indicator means are proportionally equal across groups.

**TABLE 3:** Internal consistency (α) at T1 and T1-T2 test-retest reliability (r) of the 52- and 25-item versions of the SPSI-R.

	Total SPSI-R*				English-speaking**				Spanish-speaking***			
**SPSI-R scales**	**52-item** **(α)**	**52-item** ***(r)***	**25-item** **(α)**	**25-item** ***(r)***	**52-item** **(α)**	**52-item** ***(r)***	**25-item** **(α)**	**25-item** ***(r)***	**52-item** **(α)**	**52-item** ***(r)***	**25-item** **(α)**	**25-item** ***(r)***
SPSI-R total	0.94	0.75	0.88	0.74	0.94	0.74	0.89	0.74	0.90	0.77	0.80	0.74
PPO	0.75	0.62	0.75	0.62	0.76	0.66	0.76	0.66	0.71	0.54	0.71	0.54
NPO	0.89	0.67	0.80	0.60	0.89	0.65	0.80	0.62	0.90	0.68	0.81	0.56
ICS	0.86	0.66	0.74	0.61	0.86	0.67	0.75	0.63	0.85	0.61	0.73	0.54
AS	0.82	0.63	0.83	0.61	0.82	0.66	0.84	0.65	0.79	0.54	0.81	0.52
RPS	0.94	0.64	0.82	0.64	0.94	0.67	0.83	0.66	0.93	*0.56*	0.81	0.57

AS, avoidance style; ICS, Impulsivity/Carelessness Style; n/a, not applicable; NPO, negative problem orientation; PPO, positive problem orientation; RPS, rational problem-solving; SPSI-R, Social Problem-Solving Inventory-Revised; α, Cronbach’s alpha, *r*, Pearson’s correlations. All correlations were significant at p < 0.001. * *N* = 1,069 for total sample internal consistency at T1; *N* = 279 for comparison sample T1-T2 test-retest reliability. **N = 826 for total sample internal consistency at T1; *N* = 204 for comparison sample T1-T2 test-retest reliability. ***N = 243 for total sample internal consistency at T1; *N* = 75 for comparison sample T1-T2 test-retest reliability.

**TABLE 4:** SPSI-R sensitivity to detect change in problem-solving skills over time between participants in the intervention and comparison groups.

SPSI-R version	SPSI-R scales	Intervention (PSST, *N* = 543)			Comparison (UPC/NDS, *N* = 279)			ANOVA *p-*value	Δ^2^
		**T1** ***M (SD)***	**T2** ***M (SD)***	***M*^Δ^** **T1-T2**	**T1** ***M (SD)***	**T2** ***M (SD)***	***M*^Δ^** **T1-T2**		
SPSI-R 52-items (possible range)	SPSI-R total (0–20)	13.63 (2.67)	14.58 (2.51)	0.95	13.59 (2.46)	13.73 (2.50)	0.14	<0.001	0.04
	PPO (0–20)	11.93 (3.95)	12.66 (3.92)	0.73	11.70 (3.69)	11.77 (3.68)	0.07	0.013	0.01
	NPO (0–40)	11.66 (7.68)	8.52 (6.57)	−3.14	11.85 (7.69)	11.08 (7.67)	−0.77	<0.001	0.03
	ICS (0–40)	8.34 (6.54)	7.15 (5.48)	−1.19	8.61 (6.42)	7.99 (6.31)	−0.62	0.153	0.00
	AS (0–28)	6.17 (4.89)	5.06 (4.18)	−1.11	6.24 (4.67)	6.16 (4.66)	−0.08	0.001	0.01
	RPS (0–80)	42.48 (14.75)	46.69 (15.27)	4.21	43.72 (13.37)	43.30 (13.46)	−0.42	<0.001	0.03
SPSI-R 25-items (possible range)	SPSI-R total (0–20)	12.80 (2.74)	13.66 (2.56)	0.86	13.59 (2.54)	13.79 (2.61)	0.20	<0.001	0.03
	PPO (0–20)	11.93 (3.95)	12.66 (3.92)	0.73	11.70 (3.69)	11.77 (3.68)	0.07	0.013	0.01
	NPO (0–20)	6.52 (4.13)	4.69 (3.45)	−1.83	6.72 (4.07)	6.04 (3.96)	−0.68	<0.001	0.02
	ICS (0–20)	4.57 (3.54)	3.96 (3.24)	−0.61	4.62 (3.50)	4.35 (3.40)	−0.27	0.132	0.00
	AS (0–20)	3.66 (3.76)	2.84 (3.16)	−0.82	3.72 (3.67)	3.66 (3.60)	−0.06	0.002	0.01
	RPS (0–20)	11.14 (4.12)	12.01 (4.13)	0.87	11.26 (3.79)	11.23 (3.80)	−0.03	0.001	0.01

AS, avoidance style; ICS, Impulsivity/Carelessness Style; M, mean; *M*^Δ^, difference in average score from T1-T2; NDS, non-directive support; NPO, negative problem orientation; PPO, positive problem orientation; PSST, problem-solving skills training; RPS, rational problem-solving; SD, standard deviation; SPSI-R, Social Problem-Solving Inventory-Revised; *η*^2^, partial eta squared.

## Discussion

The current study assessed the psychometric properties of 3 published versions of the SPSI-R (52-, 25-, and 10- items) in English- and Spanish-speaking caregivers of children with newly diagnosed cancer. Our findings indicate that the 52- and 25-item versions showed acceptable internal consistency for both the English and Spanish versions. This result agrees with numerous other studies using the 52- and 25-item versions of the SPSI-R (Chang, 2002; D’ Zurilla et al., 2002; Cameron et al., 2004; Hawkins et al., 2009; Jaffee and D’ Zurilla, 2009; Klein et al., 2011; Pech and O’Kearney, 2013; Wang et al., 2013; Li et al., 2016). We were not able to reproduce the 1-factor structure of the 10-item version in the current population and were therefore not able to demonstrate acceptable reliability for the 10-item version. This result is in contrast to the one other study using the 10-item version on other populations (Dreer et al., 2009). Reverse worded items are often used to reduce or eliminate acquiescence bias. The 10-item version of the SPSI-R consisted of 7 reverse worded items and 3 original positively worded items. It is known from the literature that the number and type of reverse worded items can problematically affect factor structures of measurement instruments (e.g., Zhang et al., 2016). Future research should examine the origin of the items selected for the 10-item version of the SPSI-R. Multigroup analyses showed that the factor structure of the 52- and 25-item versions of the SPSI-R are invariant across time and language, showing equal factor loadings and intercepts at two time points and for English- and Spanish-speaking caregivers. Test-retest reliability showing large correlations for the comparison groups between T1 and T2 suggest that the 52- and 25-item versions of the SPSI-R were relatively stable over time. The psychometrics of the SPSI-R 52- and 25-item versions are similar for English- and Spanish-versions. This corresponds with one other study measuring the psychometrics of the 52-item version in Hispanics (De La Torre et al., 2010), and is partly in line with a previous study that tested for factorial invariance of the 52-item version in the Spanish population (Maydeu-Olivares et al., 2000). Whereas the current study demonstrated factorial invariance across all domains of the SPSI-R, Maydeu-Olivares et al. (2000) reported invariance for all domains except for the impulsivity/carelessness scale.

The 52-, and 25-item versions of the SPSI-R were sensitive to change over time, as they both detected the expected differences in problem-solving skills between participants assigned to the intervention versus the comparison groups, except for the impulsivity/carelessness subscale. One possibility for not finding pre-post changes in this subscale is floor effects. Floor and ceiling effects can influence a scale’s responsiveness to change because they limit our ability to measure variance above or below a certain limit (Cramer and Howitt, 2004). The population broadly scored low on this scale to start and therefore there was not much space to improve on this domain. Since this domain includes items that describe impulsively making choices without thinking things through, it’s possible that there is some social desirability bias to respond low on this domain. The treatment program focuses on improving rational problem-solving, so it does make sense that we would see the greatest improvement there. Moreover, the SPSI-R 52- and 25-item versions demonstrated the same degree of differences over time for all subscales. We conclude that the 25-item version is a psychometrically reasonable substitute for the 52-item version if RPS subscales are not required. If the RPS subscales are not needed, the 25-item version can serve as a rapid assessment of problem-solving skills with less burden for the participant.

Although our findings unequivocally demonstrate strong psychometric properties of the SPSI-R, some limitations of our study should be mentioned. First, because the first two trials of the problem-solving skills intervention included only mothers, this population was overrepresented. Future studies are needed to determine whether the psychometrics of the SPSI-R are robust for fathers. Because of the limited number of fathers in our sample, we were not able to test for factorial invariance across gender in this population. Second, our findings are limited to caregivers of children with cancer. Future work needs to expand our findings with additional groups to determine generalizability.

In conclusion, we have demonstrated that the 52- and 25-item versions of the SPSI-R are psychometrically sound measures to assess problem-solving ability in both English and Spanish caregivers of children with cancer. The 25-item version is a reasonable substitute for the 52-item if the subscales of the RPS are not required. The 10-item version of the SPSI-R is not a psychometrically sound substitute for the longer versions of the SPSI-R either for assessment of change or for screening problem-solving skills.

## Data availability statement

The raw data supporting the conclusions of this article is confidential data and can be made available upon reasonable request.

## Ethics statement

The studies involving humans were approved by the Internal Review Board of the University of Rochester School of Medicine and Dentistry. IRB protocol numbers: RSRB 05736 2002 trial; RSRB 09840 2005 trial; RSRB 38174 2013 trial. The studies were conducted in accordance with the local legislation and institutional requirements. The participants provided their written informed consent to participate in this study. Written informed consent was obtained from the individual(s) for the publication of any potentially identifiable images or data included in this article.

## Author contributions

SS: conceptualization, methodology, writing original draft, formal analysis, and visualization. SP, RN, and OS: funding acquisition, conceptualization, methodology, data collection, supervision, and writing review and editing. KD: conceptualization, methodology, data collection, supervision, resources, and writing review and editing. DF: methodology, formal analysis, validation, and writing review and editing. MD, KI, NS, and MA: conceptualization, methodology, data collection, supervision, and writing review and editing. MV: data collection, project administration, and writing review and editing. All authors contributed to the article and approved the submitted version.
